# Exploring aquaporin functions during changes in leaf water potential

**DOI:** 10.3389/fpls.2023.1213454

**Published:** 2023-08-08

**Authors:** Caitlin S. Byrt, Rose Y. Zhang, Isobel Magrath, Kai Xun Chan, Annamaria De Rosa, Samantha McGaughey

**Affiliations:** Division of Plant Sciences, Research School of Biology, College of Science, Australian National University, Acton, ACT, Australia

**Keywords:** hydraulic, solute flux, water channel, membrane transport, hydration

## Abstract

Maintenance of optimal leaf tissue humidity is important for plant productivity and food security. Leaf humidity is influenced by soil and atmospheric water availability, by transpiration and by the coordination of water flux across cell membranes throughout the plant. Flux of water and solutes across plant cell membranes is influenced by the function of aquaporin proteins. Plants have numerous aquaporin proteins required for a multitude of physiological roles in various plant tissues and the membrane flux contribution of each aquaporin can be regulated by changes in protein abundance, gating, localisation, post-translational modifications, protein:protein interactions and aquaporin stoichiometry. Resolving which aquaporins are candidates for influencing leaf humidity and determining how their regulation impacts changes in leaf cell solute flux and leaf cavity humidity is challenging. This challenge involves resolving the dynamics of the cell membrane aquaporin abundance, aquaporin sub-cellular localisation and location-specific post-translational regulation of aquaporins in membranes of leaf cells during plant responses to changes in water availability and determining the influence of cell signalling on aquaporin permeability to a range of relevant solutes, as well as determining aquaporin influence on cell signalling. Here we review recent developments, current challenges and suggest open opportunities for assessing the role of aquaporins in leaf substomatal cavity humidity regulation.

## Introduction

Crop productivity is directly impacted by water availability which is influenced by the climate (Cook et al., 2018; Tabari, 2020). Our climate is impacted by levels of carbon in the atmosphere (412 ppm), which is now 47% higher than it was at the beginning of the industrial age (280 ppm; Buis, 2019). The carbon in the atmosphere traps heat and with every degree increase in heat the atmosphere can hold 7% more water (Trenberth, 2011). Water molecules tend to adhere to each other which means higher atmospheric water holding capacity is problematic. Rather than agricultural areas experiencing ideal conditions, such as fairly evenly distributed and consistent moderate rainfall, we are instead facing a future of increasingly frequent and extreme weather events, like floods and droughts (Holden, 2019).

Both too much and too little rain limits crop productivity. Crops submerged in water can become oxygen limited restricting respiration and limiting productivity, and when water loss from leaves exceeds root water uptake then plant net hydration decreases which can lead to loss of turgor and hydraulic failure (Bailey-Serres and Voesenek, 2010; Bartlett et al., 2012). Plant tissue hydration needs to be carefully coordinated (Meinzer et al., 2009). Limited water availability triggers leaf stomatal closure to reduce transpiration and limit loss of hydration in plant tissues. In addition to stomatal closure, changes in leaf cell membrane water flux occur to regulate plant tissue hydration (Wong et al., 2022). The progressive reduction in water flux through mesophyll cell aquaporins was suggested by Wong et al. (2022) to be involved in maintaining cytosolic water potential when leaves experience declining humidity in the air. Aquaporins are tetrameric membrane intrinsic proteins renowned for controlling membrane water flux (Maurel et al., 2015). Both plasma membrane and tonoplast localised aquaporins have been reported to influence leaf hydration and hydraulic conductance in crops such as barley (*Hordeum vulgare*) and *Sorghum bicolor* (Schley et al., 2022; Sharipova et al., 2022).

Resolving how aquaporin functions influence leaf substomatal cavity humidity is an important step towards investigating ways to adapt crops to conditions with sub-optimal water availability (Verslues et al., 2023). All plants have aquaporins, they are abundant in cell membranes surrounding leaf veins, mesophyll and stomata and they influence the flux of water into and out of these cells and the leaf air space. The objective of this review is to highlight recent developments in studying plant regulation of leaf humidity and share examples of factors for consideration when investigating the role of aquaporins in influencing solute flux in leaf cells relevant to maintaining tissue humidity. We provide information about key challenges relevant to studying aquaporin functions and outline options for researchers who are working towards progressing our knowledge of how aquaporins influence leaf humidity regulation.

## Water availability and leaf vapour pressure variation

Soil and atmospheric aridity restrict plant productivity (Dietz et al., 2021). Insufficient water in the environment can rapidly translate to insufficient water inside plants (Hu et al., 2022). The vapour pressure in plant leaves is not always saturated, instead the intercellular relative humidity in leaves varies in response to environmental changes and it can be regulated by the plant (Cernusak et al., 2018). Plants can adjust the water potential in their leaves to sustain a range of different levels of humidity in the intercellular mesophyll air space, for example from 80 to 97% (Wong et al., 2022). As plants transpire water moves from leaf cells to the air space within the leaf where it exits the leaf *via* stomata. The humidity gradients in leaf mesophyll cells influence the vapour flux to the stomatal pores. When there is maximum possible humidity in the leaf it is considered to be saturated. The relative level of saturation across the leaf can change and it can respond to changes in the external humidity in the air outside the leaf.

**Figure 1:**
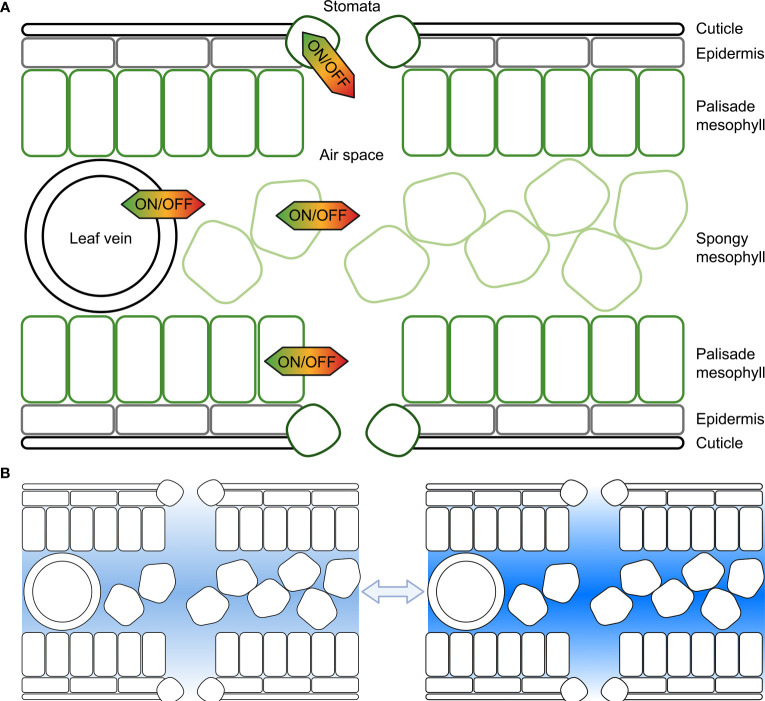
Representation of the potential for cell membrane water flux to be tuned at different cellular locations within the leaf to influence substomatal cavity humidity. **(A)** Each cell, including cells forming the leaf epidermis, palisade mesophyll cells, spongy mesophyll cells or cells surrounding leaf veins, or guard cells in the stomata can regulate the water flux across their plasma and tonoplast membranes, and other organelle membranes; **(B)** The humidity within leaf air spaces changes in response to environmental conditions, and cell signaling influences membrane water flux, here lighter shading represents lower humidity and darker shading represents higher humidity. Previous studies have reported values of relative humidity inside leaves varying from as low as 70% up to saturated; plants can modify leaf air space humidity by regulating the flux of water that moves in and out of veins and leaf mesophyll cells (following Wong et al., 2022).

When the air outside a leaf becomes increasingly dry the difference in vapour pressure between the outside air and the inside of the leaf increases, and the saturated front retreats from the substomatal cavity towards the intercellular space creating a longer pathlength for the water flux and increasing the resistance (Wong et al., 2022). The water potential in the cell walls or intercellular space between the leaf cells may sit at equilibrium with the relative humidity in the substomatal cavity whilst the intracellular cell cytoplasm water potential can differ relative to the humidity in the substomatal cavity creating a pressure difference between the cytosol/intracellular space and cell wall/intercellular space. Leaf cells control the flow of water from the intracellular to the intercellular space (**Figure 1**), which is achieved by controlling plasma membrane and tonoplast hydraulic conductivity. There are of course many other factors that influence leaf humidity, such as leaf anatomy and architecture, leaf size, opening of stomata, vein arrangement and the influence of transpiration.

## Membrane flux and leaf humidity

Cell membrane hydraulic conductivity is influenced by the lipid composition of the membrane, the protein composition in the lipid membrane and the regulation of membrane protein functions in the lipid membrane (Mamode Cassim et al., 2019). There are a range of factors that influence plant cell membrane permeability. For instance, different plants, plant tissues and cell types can have diverse cell membrane lipid compositions which can influence the permeability of the membrane and the conformation of membrane proteins (Schuler et al., 1991; Martínez-Ballesta and Carvajal, 2016; Mamode Cassim et al., 2019). Plant cell membranes are packed with membrane proteins, and the abundance and type of membrane proteins influences the permeability of the membrane (Chrispeels et al., 1999).

Of the many types of proteins in cell membranes there are a range of different mechanisms involved in diverse metabolic, signaling and transport functions. The proteins involved in solute transport functions can be regulated to dynamically influence the cell membrane permeability. The regulation of aquaporins in particular influences the hydraulic conductivity of cell membranes (Maurel, 1997; Maurel et al., 2015). Recently the progressive reduction of mesophyll cell aquaporin water flux was reported to be a candidate mechanism involved in maintaining cytosolic water potential when leaves experience declining humidity in the air (Wong et al., 2022). Investigating the contribution of aquaporins to the regulation of leaf airspace humidity is important because it is relevant to determining how to adapt crops for water limited environments.

## Aquaporin functional diversity and regulation

Aquaporins are pore-forming membrane proteins that are part of the Major Intrinsic Protein (MIP) family. Many but not all types of aquaporins can function as water channels (Tyerman et al., 2021). Aquaporin contributions to tissue hydraulics is known to influence plant transpiration; they can influence solute uptake in roots, root-to-shoot hydraulic conductance, cellular osmolarity maintenance, cell-to-cell solute flux and stomatal regulation (Vera-Estrella et al., 2004; Moshelion et al., 2015; Maurel et al., 2016; Israel et al., 2021; Patel and Mishra, 2021). Aquaporin function is important in relation to plant maintenance of cytosolic osmolarity when adjusting to environmental stresses (Liu et al., 2006), and it has been suggested that aquaporins may be implicated in foliar water uptake to overcome drought stress (Vignesh and Palanisamy, 2021).

The plant Kingdom has the greatest aquaporin diversity compared to the animal, fungi, eubacterial, archeabacterial or protista Kingdoms. For example, there are 121 aquaporin isoforms in some *Brassica* species (Yuan et al., 2017), whereas humans and many other mammals have 13 different aquaporins, and many single-celled organisms have one or two different aquaporins (Tanghe et al., 2006; Verkman, 2008; Tesan et al., 2021). Arabidopsis has 35 aquaporins, and different aquaporins are up or down regulated in response to varying environmental conditions in different cells and tissues (Jang et al., 2004). In higher plants, aquaporins fall into five subfamilies categorised as Plasma membrane Intrinsic Proteins (PIPs), Tonoplast Intrinsic Proteins (TIPs), NOD26-like intrinsic proteins (NIPs), small basic intrinsic proteins (SIPs), and uncharacterised intrinsic proteins (XIPs) (Danielson and Johanson, 2008). Presumably it was useful for plants to duplicate and diverge the number and types of aquaporin genes, respectively, as part of adapting to different terrestrial environments. Major rearrangements were reported in the aquaporin gene families in seagrasses relative to terrestrial grasses which is indicative of the importance of aquaporins for plant adaptation to environments where low humidity limits water availability as distinct from the submerged aquatic environments where seagrasses are found (Olsen et al., 2016).

Diverse functions have been attributed to the many different types of plant aquaporin isoforms, and a single type of aquaporin protein can have multiple functions such as transporting several solutes (Tyerman et al., 2021; Groszmann et al., 2023). Aquaporins can transport water, gasses like carbon dioxide and oxygen, uncharged molecules such as urea and glycerol, nutrient/metal/mineral ions, organic molecules (e.g. aluminum malate) and signaling molecules (e.g. hydrogen peroxide, H_2_O_2_) (Maurel et al., 2015; Wang et al., 2017b; Tyerman et al., 2021). Aquaporins are one of the most abundant proteins observed in plant membranes, but any given membrane can have multiple types of aquaporins present and each aquaporin type can have different functions and can potentially be regulated independently.

**Figure 2:**
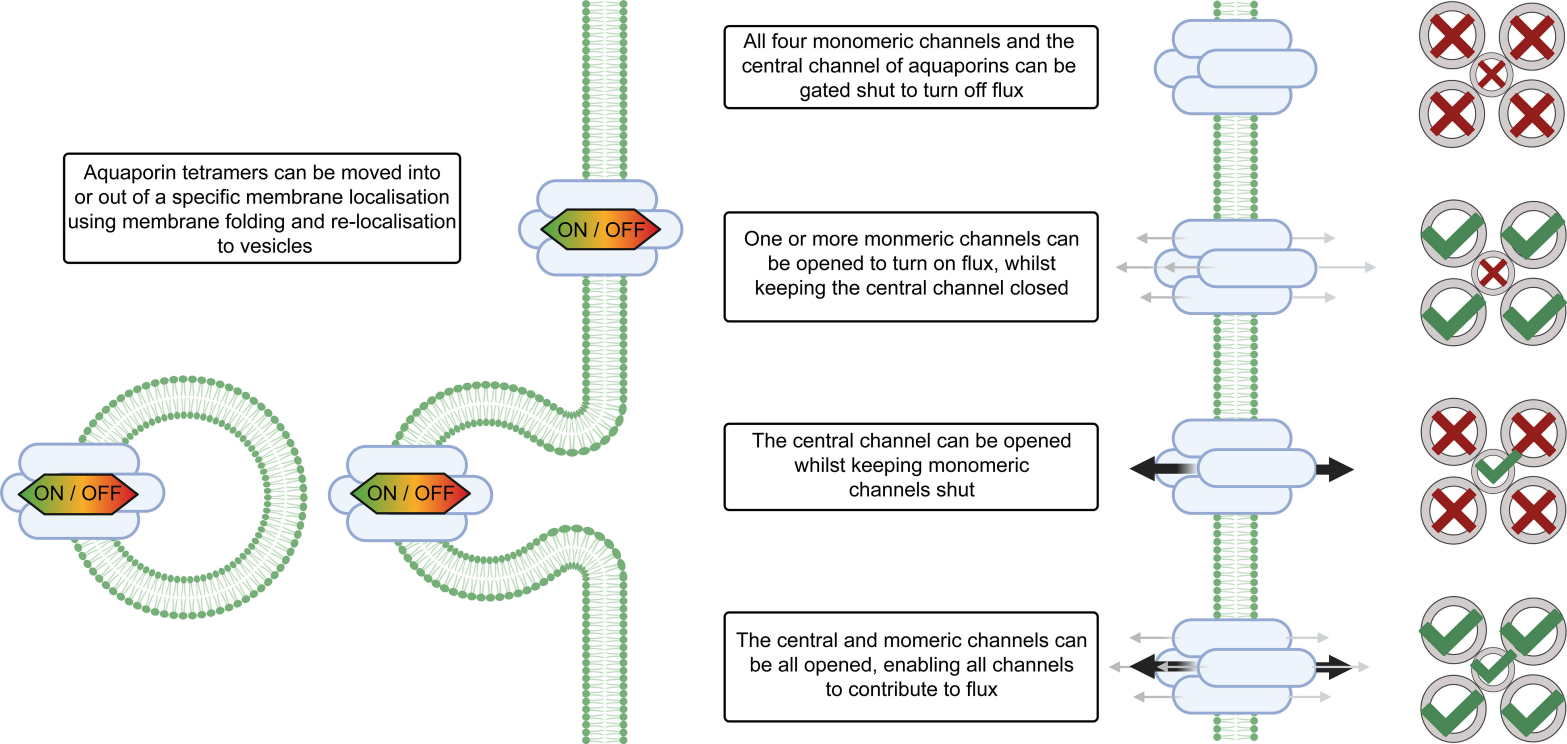
Aquaporin localisation and gating influences solute flux across cell membranes. The magnitude of water flux through the aquaporins in cell membranes can be controlled by changing the abundance of different aquaporins in the cell membrane, altering the localisation of aquaporins within the cell, changing the stoichiometry of aquaporin tetramers, and gating of aquaporin channels *via* signaling and post-translational modifications. The stoichiometry of the aquaporin refers to which types of monomers are present in the aquaporin tetramer. Aquaporins tetramers have four monomeric channels and a central channel in the middle and all five of the channels can be gated independently. Note that differential gating of individual monomers is not represented in this figure, for additional examples see Tyerman et al. (2021). For some types of aquaporins the monomeric channels may be only permeable to water (represented by grey arrows), and the central channel may be permeable to other molecules (represented by black arrows) (Yu et al., 2006).

Plants evolved a multitude of processes to ensure coordinated regulation of aquaporin functions and by extension there are many levels of regulation of cell membrane permeability (**Figure 2**). Consideration of aquaporin regulatory features is important when deciphering their involvement in regulating tissue water content. Examples of aquaporin regulatory processes include: regulation of aquaporin gene transcription level and translation, and regulation of the complexing of aquaporin monomers into tetramers, i.e. aquaporin stoichiometry which influences tetramer function (Zelazny et al., 2007; Fox et al., 2017). Aquaporins may also be subject to a range of other post-translational regulatory mechanisms such as oxidation and sulfonation, and the full diversity of modifications of aquaporins that occurs post-translation is yet to be resolved (Kamath et al., 2011; Nesverova and Törnroth-Horsefield, 2019). Aquaporin localisation in cellular membranes influences membrane permeability, with aquaporin function and localisation being able to rapidly change in response to cellular signals/environmental stresses (Hosy et al., 2015). Examples of types of cell signals that are relevant to aquaporin regulation include: abscisic acid, calcium, cyclic nucleotides, ethylene, pH, reactive oxygen species, salicylic acid (Chen et al., 2013; Kapilan et al., 2018; McGaughey et al., 2018). Recently the jasmonic acid analog coronatine (COR) was also reported to influence aquaporin function; COR induced *Zea maize PIP2;5* expression and interacted with ZmPIP2;5 through binding potentially influencing water uptake during stress (He et al., 2022).

Aquaporin permeability and conductance is regulated by channel gating and by a range of post-translational modifications (Törnroth-Horsefield et al., 2010). This means that aquaporins can be turned on and off, somewhat like a tap, and plants have many options for controlling when and where those taps are turned on and off, and also for controlling which solutes flow through those taps. Plant aquaporin regulation is a key part of adapting to environmental stresses, such as changing humidity (Kapilan et al., 2018). Plants tune aquaporin functions in different cells and tissues to adjust diurnal leaf hydraulics, to adapt to water deficit and to adjust growth towards surviving drought (Prado et al., 2019; Zhang et al., 2019; Patel and Mishra, 2021). Leaf cells such as the vascular bundle sheath and mesophyll cells have been reported to influence leaf water balance (Attia et al., 2020), but the differential regulation of aquaporin-facilitated membrane solute flux that occurs in the different cell types in plant leaves, such as in mesophyll relative to bundle sheath cells, is yet to be fully resolved (Shatil-Cohen et al., 2011).

In rice (*Oryza sativa*) cell-specific comparison of aquaporin encoding gene expression levels in mesophyll cells, bundle sheath cells and vascular tissue cells revealed that the sets of aquaporin PIP, TIP, NIP and SIP isoforms that were most highly expressed in these different cell types varied (see Figure 2 of Hua et al., 2021). For example, transcript levels of *OsPIP2;7, OsTIP4;3* and *OsTIP4;1* were notably high specifically in mesophyll cells, and the expression *OsPIP2;6* and *OsPIP2;7* expression was higher in both mesophyll and bundle sheath cells than in vascular cells; *OsTIP1;1*, *OsTIP1;2*, *OsPIP2;3, OsPIP1;2, OsPIP2;2, OsTIP2;2, OsPIP1;1* and *OsPIP1;3* expression was notably greater in bundle sheath cells than mesophyll and vascular cells; and the four most highly expressed aquaporins in the vasculature were *OsPIP2;4, OsNIP2;2, OsSIP2;1* and *OsNIP2;1* (Hua et al., 2021). It is likely that coordination of solute flux across these different cell types is achieved through interaction of these different aquaporin isoforms with each other and interactions with other types of proteins that influence aquaporin roles in solute flux. For example, a zinc finger transcription factor called DROUGHT AND SALT TOLERANCE (DST) regulates a leucine-rich repeat receptor-like kinase named *Leaf Panicle 2* which can interact with OsPIP1;1; OsPIP1;3 and OsPIP2;3 and can influence drought sensitivity in rice (Wu et al., 2015).

**Figure 3:**
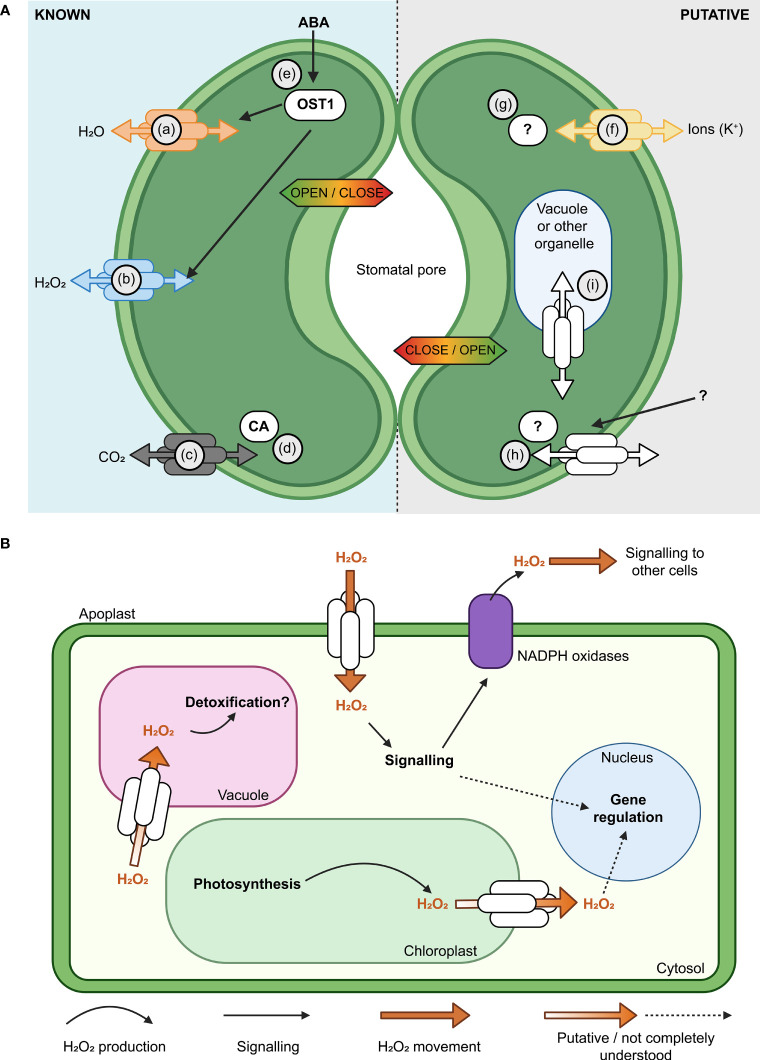
Aquaporins have multiple roles in guard cell (GC) membrane transport and signaling. **(A)** Modulation of the activity of aquaporins influences the aperture of stomata (Hachez et al., 2017). Known (left, guard cell 1) and putative (right, guard cell 2) examples of aquaporin roles in GC are represented, such as; (a) Aquaporin functions in regulating H_2_O flux across the plasma membrane (PM) (Bienert and Chaumont, 2014; Maurel et al., 2016); (b) Some aquaporins, such as AtPIP2;1, influence hydrogen peroxide (H_2_O_2_) flux; (c) Some aquaporins have roles in carbon dioxide (CO_2_) transport and can interact with carbonic anhydrase (CA; d), which converts between CO_2_ and HCO_3_^-^ (Wang et al., 2016; Groszmann et al., 2017; Zhang et al., 2018); (e) Water limitation can trigger increased abscisic acid (ABA) in plants, ABA influences Open Stomata 1 (OST1) kinase and the regulation of multiple proteins, including AtPIP2;1 (Grondin et al., 2015; Maurel et al., 2016); (f) Subsets of aquaporins can transport monovalent ions like potassium (K^+^; Byrt et al., 2017); (g)> Post-translational modifications (PTMs) such as phosphorylation/de-phosphorylation can switch some aquaporins between functioning more as ion channels rather than water channels (Qiu et al., 2020); AtPIP2;1 C-terminal (serine 280/283) PTMs influence water and transport, leaf hydraulics (involves 14-3-3 proteins, Prado et al., 2019) and light induced stomatal opening (Huang et al., 2020). The complement of PTMs, kinases and phosphatases involved are yet to be reported; (h) Further research is needed to resolve the complement of external signals, receptors and molecular components involved in signaling cascades that influence aquaporin function in GCs;(i) Knowledge gaps remain in our understanding of how tonoplast and PM aquaporin fluxes are coordinated. **(B)** Subsets of aquaporins have roles in influencing H_2_O_2_ flux across various cell membranes (plasma membrane, chloroplast envelope, tonoplast), they can influence H_2_O_2_ flux from chloroplasts to the cytosol and vacuole and they are likely to be part of more extensive signaling networks.

Plant aquaporins have multiple roles in guard cells, some involve transport of water and other molecules (Maurel et al., 2016; **Figure 3**). Aquaporin function was reported to influence hydrogen peroxide (H_2_O_2_) entry into guard cells which then impacts the influence of abscisic acid signaling triggering stomatal closure through Open Stomata 1 (OST1) protein kinase mediated phosphorylation (Grondin et al., 2015; Rodrigues et al., 2017; Cui et al., 2021). In Arabidopsis differential phosphorylation of the C-terminal of AtPIP2;1 was shown to affect light induced stomatal opening (Huang et al., 2020), and in *Zea maize* PIP2;5 was shown to be involved in the transpiration decrease observed under high vapour pressure deficit (VPD) (Ding et al., 2022). There are sub-sets of aquaporins that have both ion and water channel functions and these aquaporins could potentially be involved in influencing the guard cell membrane ion flux, such as K^+^ flux, that is part of stomatal movement (MacRobbie, 2006; Byrt et al., 2017; Durand et al., 2020; Qiu et al., 2020). It may be possible in the future to optimise stomatal dynamics, transpiration and leaf humidity by manipulating aquaporin water, ion and H_2_O_2_ transport functions (Wang et al., 2017a; Ding and Chaumont, 2020).

Environmental stresses such as high VPD can cause H_2_O_2_ to accumulate within and outside of plant cells (Munné-Bosch et al., 2001; Ding et al., 2022). H_2_O_2_ is required as a cellular signal in various plant processes including stomatal closure, which is a rapid and direct response to high VPD. However, over-accumulated H_2_O_2_ can be damaging to the plant cell due to its high redox reactivity. Consequently, the mechanism(s) by which H_2_O_2_ is transported within and between cells for signalling and detoxification are of significant interest for improving crop abiotic stress tolerance (Zhang et al., 2023). Some aquaporins can transport H_2_O_2_ when expressed in heterologous expression systems indicating that these aquaporins have the potential to influence H_2_O_2_ transport *in planta* (Hooijmaijers et al., 2012; Groszmann et al., 2023; **Figure 3B**). The plasma membrane-localised PIP2;1 aquaporin facilitates movement of apoplastic H_2_O_2_ produced by NADPH oxidases across the plasma membrane into the cytosol. PIP2;1-mediated H_2_O_2_ transport is actively regulated *via* phosphorylation by multiple proteins including Open Stomata 1 (OST1) and Brassinosteroid insensitive 1-associated receptor kinase 1 (BAK1) (Grondin et al., 2015; Rodrigues et al., 2017). Perturbing function or phosphorylation of *pip2;1* and *ost1* disrupts stomatal closure and other whole-plant responses reliant on H_2_O_2_ movement such as systemic acquired acclimation (Rodrigues et al., 2017; Fichman et al., 2021). These results demonstrate the importance of aquaporin-mediated H_2_O_2_ fluxes to plant acclimation to environmental stresses.

In contrast to movement of extracellular H_2_O_2_, relatively less is known about intracellular H_2_O_2_ movement. It is possible that some aquaporins could contribute to influencing sequestration of excess H_2_O_2_ into the vacuole (Wudick et al., 2015). However, whilst this hypothesis is attractive, testing and experimental evidence are still needed (Smirnoff and Arnaud, 2019). H_2_O_2_ produced in chloroplasts can be exported to the nucleus in response to high light stress or viral infection, presumably to fulfil signalling functions (Caplan et al., 2015; Exposito-Rodriguez et al., 2017; Mullineaux et al., 2020; Phua et al., 2021). H_2_O_2_ export was detected from isolated chloroplasts exposed to high light stress, and this export was blocked by the addition of the aquaporin inhibitor acetazolamide (Borisova et al., 2012). Intriguingly, three TIP aquaporins were recently localised to chloroplasts (Beebo et al., 2020); one of which has been reported to transport H_2_O_2_ when expressed heterologously in yeast (Hooijmaijers et al., 2012). Collectively, these observations reveal the possibility for organellar aquaporins to have roles in regulating and distributing H_2_O_2_ within cells. Nevertheless, rigorous future investigation is required to validate this speculative hypothesis. There are also several knowledge gaps related to aquaporin involvement in transporting H_2_O_2_. For example, mitochondria and peroxisomes are also sources of H_2_O_2_ (Phua et al., 2021), and further research is required to elucidate potential aquaporin functional roles in these organelles. Recent studies identified six Arabidopsis PIP and TIP aquaporin isoforms in the inner mitochondria membrane *via* proteomics (Møller et al., 2020), and GFP-tagging of AtTIP5;1 revealed examples of localisation in the mitochondria of pollen tubes (Soto et al., 2010), however there are no confirmed aquaporins that are targeted specifically to the peroxisome. Furthermore, the identity of cellular protein(s) targeted by aquaporin-transport of H_2_O_2_ for signaling purposes is still unclear. Nevertheless, since many H_2_O_2_-responsive proteins are involved in major cellular processes such as RNA metabolism (Huang et al., 2019), manipulation of H_2_O_2_ movement within leaf cells during stresses such as water deficit may be a promising approach for improving crop productivity during drought.

## Testing aquaporin roles in influencing leaf humidity

Plant adaptation to different environmental conditions can require adjustment to leaf water potential and transpiration. When a change in leaf water potential is needed, changes in cell chemical and hormonal signaling, membrane potential and pressure can be detected (Davies et al., 2002; Osakabe et al., 2014). Examples of the types of signaling changes include changes in abscisic acid signaling, calcium signaling, cyclic nucleotide signaling, reactive oxygen species signaling, and changes in pH and regulation of kinases and phosphatases (Gilroy et al., 2014; Sewelam et al., 2016). Each of these types of signaling changes can influence the regulation of aquaporins at multiple levels (Li et al., 2011; McGaughey et al., 2018). Signaling pathways can influence the level of transcription of aquaporin genes, influence the stoichiometry of aquaporin tetramers, change the abundance of aquaporin proteins, change the localisation of aquaporin proteins, change aquaporin interactions with other proteins and change the gating of aquaporin proteins (Fetter et al., 2004; Maurel et al., 2015; Fox et al., 2020). Changes in aquaporin gating can influence whether the monomeric channels and central channel formed in the middle of the aquaporin tetramer are open or closed and influence their permeability to a range of different types of molecules (Qiu et al., 2020).

**Figure 4:**
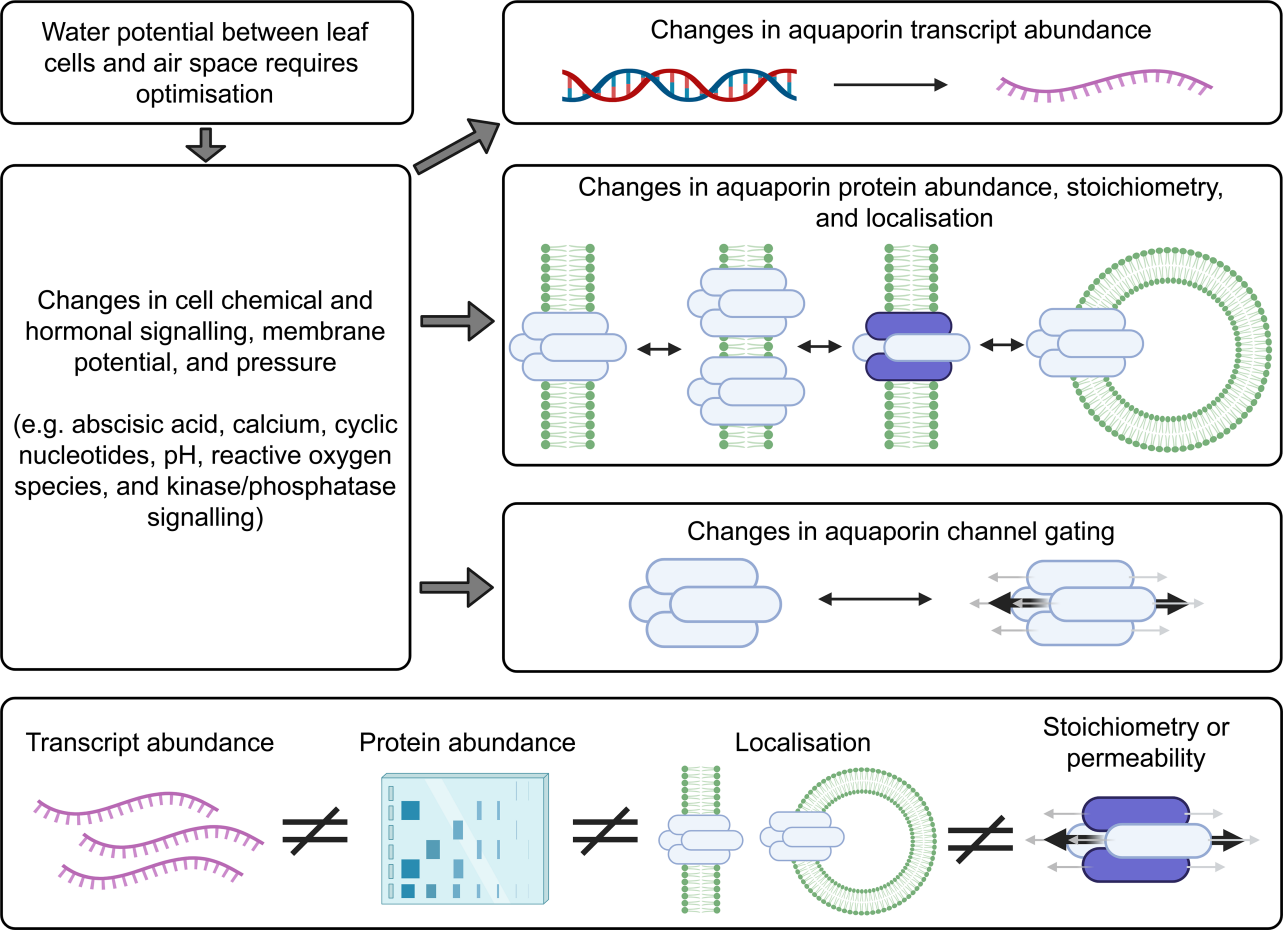
Factors to consider when investigating aquaporin contributions to cell biology. Changes in the abundance of many different types of aquaporins may occur following changes in environmental conditions. The localisation of aquaporins can change and the tetramer stoichiometry can change, for example aquaporins can move around in small vesicles. Aquaporin tetramers have four monomeric channels and a central channel, and all five of these changes can be gated. There is capacity for the different levels of aquaporin regulation to be regulated independently. Information about aquaporin gene transcript abundance (represented in purple) is not a suitable proxy for assessing protein abundance, they are not equivalent measures, and the total abundance of aquaporins in a sample extracted from plant tissues does not give any indication of the cellular or subcellular localisation of the aquaporin or the aquaporin stoichiometry, or protein-protein interaction status or state of gating (Fox et al., 2017). This means that it is insufficient to just test for aquaporin transcript levels, or only measure the total amount of aquaporin protein in the tissue, and it is important to test for aquaporin subcellular localisation and determine aquaporin stoichiometry, protein interactions and state of gating when assessing the extent to which aquaporins are contributing to cell membrane permeability (Maurel, 2007; Maurel et al., 2015).

The dynamics of aquaporin regulation are tissue, cell and organelle specific (Cui et al., 2021). Transcriptional and post-translational levels of regulation of aquaporins and cell type-specific regulatory features all need to be considered when investigating aquaporin contribution to plant hydraulics. This means that consideration needs to be given to multiple layers of regulation such as mRNA levels, subcellular localisation of the proteins and gating of the protein channels (**Figure 4**). Standard approaches to studying membrane proteins, which are also relevant to studying aquaporin roles in plant hydraulic regulation, were previously summarised by Tang et al. (2020), these included: (1) Identification of candidate proteins from omics data such as whole genome sequencing, ionomic profiling, genome-wide association studies and analyses based on transcriptomic and proteomic data sets; (2) *in silico* analysis such as homology-based genomic analysis, RNA-sequencing analysis and quantitative proteomic analysis for predicting function; (3) Testing membrane protein function using systems such as proteoliposomes, living cell-based heterologous systems, electrophysiological approaches and use of fluorescent biosensors; and (4) functional assessment *in planta* using expression pattern analysis, testing protein subcellular localisation; phenotyping plants with loss of protein function like T-DNA mutants or RNAi mutants or plants with either loss of function or enhanced expression; for example approaches like CRISPR-Cas9 (Miki et al., 2021) or TALENs (Beurdeley et al., 2013) have been used to modify the plant genome to influence the regulation of a given target mechanism of interest.

**Figure 5:**
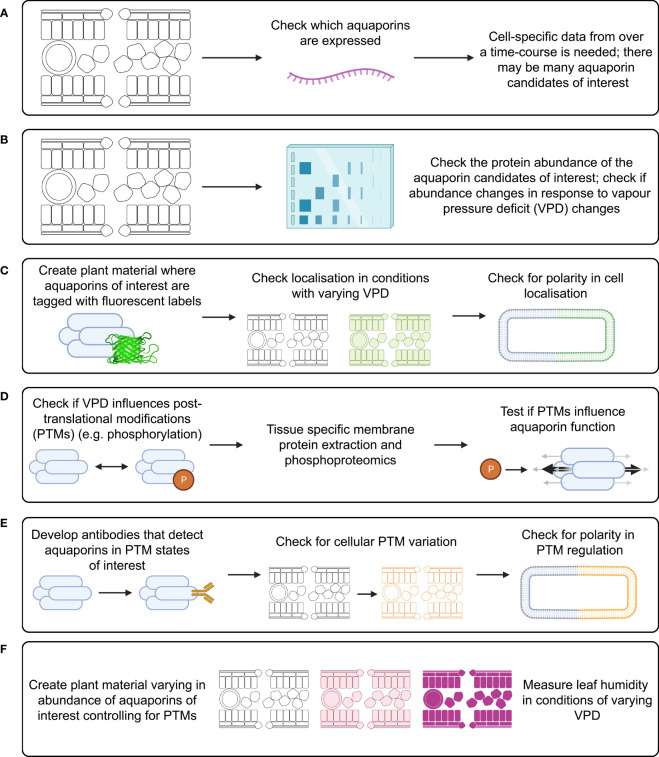
Investigation of aquaporin influence on substomatal cavity humidity requires study of many factors that can influence aquaporin function. Investigation of each of these factors needs to be cell-type specific and over a time-course following changes in VPD, examples of approaches include: **(A)** measuring aquaporin transcript abundance; **(B)** measuring aquaporin protein abundance; **(C)** checking for subcellular localisation of aquaporin proteins; **(D)** checking what post translational modifications (PTMs) occur *in planta* and how PTMs influence aquaporin function; **(E)** checking how relevant aquaporin PTMs influence localisation; and **(F)** studying leaf humidity in plant material with modified aquaporin function and regulation, such as aquaporin loss-of-function lines, lines over-expressing aquaporins, and potentially lines where aquaporins are edited to alter their potential for PTMs.

Aquaporin protein abundance does not always scale with mRNA levels (Buccitelli and Selbach, 2020). The regulation of aquaporin mRNA transcript levels and the abundance of the proteins they encode has sometimes been observed to be linked, but they have also been observed to be regulated independently (Marulanda et al., 2010; Muries et al., 2011; Stanfield and Laur, 2019; Jiménez et al., 2022). This means there is not necessarily a consistent relationship between transcript level changes and cell membrane aquaporin protein abundance changes (Fox et al., 2017). The following examples were derived from studies of aquaporin regulation in roots rather than leaves but the principles and lessons from these studies apply to all plant tissues: In response to salt stress Arabidopsis roots upregulate and downregulate multiple different aquaporin transcripts, and the trends in different root zones, cell types, and times after stress application vary depending on growing conditions (Luu and Maurel, 2013; McGaughey et al., 2018). Arabidopsis roots alter the sub-cellular location of aquaporins in root cell membranes in response to environmental change and signals, such as salt stress and reactive oxygen species stress signaling (Luu et al., 2012; Wudick et al., 2015; Ueda et al., 2016). Regulation of aquaporin function has been reported to involve changes in protein phosphorylation that influence aquaporin localisation and function (Prak et al., 2008; Vialaret et al., 2014; Pou et al., 2016; Qiu et al., 2020). Even within one single cell the aquaporins can be regulated differently at different sites within the membrane. For example, previous studies have shown that polar localisation of aquaporins on a particular section of cell membrane can be important for controlling hydraulics, such as where aquaporin regulation is important for lateral root emergence (Péret et al., 2012). When sampling plant tissues to investigate aquaporin regulation it is important to note that even just the act of cutting a plant can change aquaporin regulation and hydraulic conductivity, and changes in light cause differential regulation of aquaporins (Prado et al., 2013; Meng et al., 2016). With these and other complexities in mind, a list of options for methodological approaches that are relevant to further investigating aquaporin contributions to influencing leaf substomatal cavity humidity were collated (**Figure 5**).

There are likely to be many aquaporins involved in regulating the flux of water in and out of leaf cells, and different aquaporins would be expected to have different roles in different leaf cell types. This means that leaf transcriptome analysis derived from data where whole leaves were sampled is unlikely to reveal which specific aquaporin transcript changes are relevant to regulating leaf water potential. To narrow down which aquaporins may be most of interest it would be applicable to use cell-specific RNA-seq analysis involving laser dissected leaf epidermal, mesophyll, vasculature and guard cells, similar to the approach taken by Berkowitz et al. (2021) except that instead of using chemical stresses the treatment would involve changing the air VPD (**Figure 5A**). The approach by Bellati et al. (2016) was focused on analysis of aquaporin roles in roots in response to osmotic and oxidative stress treatments, they used immuno-purification coupled to protein identification and quantification by mass spectrometry. This type of approach for aquaporin quantification is ideal because it is difficult to get specific antibodies for different aquaporin isoforms due to the high similarity in aquaporin amino acid sequences, which limits options for using Western Blots to assess protein abundance (Hachez et al., 2006). If a similar approach was applied to assess aquaporin roles in leaves then ideally it would be cell-specific and represent a time-course after changes in VPD were applied (**Figure 5B**). Leaf cells would need to be harvested over a time-course to account for the potential for aquaporin-encoding gene transcript abundance trends to vary depending on the time after treatment (**Figure 5A**). Capture of temporal transcriptomic recordings of single leaf cells following VPD changes would assist in identifying the number of different candidates likely to be implicated in adapting to changes in VPD (Chen et al., 2022). The methods and approach that were used by Uhrig et al. (2021) to assess diurnal dynamics of the Arabidopsis rosette proteome and phosphoproteome are also relevant because studying the phosphorylation of aquaporin proteins is important for resolving their function and influence on tissue hydraulics (Prado et al., 2019).

The proteomics approach by Uhrig et al. (2021) enabled the quantification of nearly 5000 proteins and 800 phosphoproteins, and Yavari et al. (2022) recently quantified 9120 proteins in Arabidopsis leaves in a study assessing responses to different light treatments. The proteins quantified included many aquaporins such as PIP1;1, PIP1;3, PIP1;4, PIP1;5, PIP2;1, PIP2;3, PIP2;6, PIP2;7, TIP1;1, TIP1;2, TIP2;2, NIP6;1 and SIP1;1 (see supplemental File S2B of Yavari et al., 2022). Previous studies reported data indicating that PIP1, PIP2 and TIP type aquaporins can influence leaf hydraulic conductance and leaf water content in model species and crops (Katsuhara et al., 2003; Da Ines et al., 2010; Postaire et al., 2010; Prado et al., 2013; Schley et al., 2022). The advancement in nanoscale phosphoproteomics approaches may provide avenues in the future for determining single-cell candidate aquaporin protein abundance and phosphorylation state (Birnbaum et al., 2022; Tsai et al., 2023).

Tagging candidate aquaporins of interest using fluorescent markers and expressing them in plants can enable assessment of the localisation of the target aquaporins in different tissues and cells, including assessment of whether the localisation is around the entire cell membrane, internalised or in a polar pattern (**Figure 5C**) (Tian et al., 2004; Li et al., 2011). The tagged proteins need to be expressed using their native promoter to preserve native targeting signals, and controls included to check whether the tag influences the results. It is relevant to determine how post-translational modifications of aquaporins influence both their localisation and their osmotic permeability and permeability to other molecules (see Wu et al., 2015) (**Figure 5D**). Examples of phosphatases and kinases that have been reported to either interact with or are candidates for influencing the function of plant aquaporins are included in **Tables 1**, **2**. To explore the function of different PTM states for specific candidates the candidates can be expressed and tested in heterologous expression systems (Qiu et al., 2020). If antibodies can be generated that detect aquaporins in different phosphorylation states then these can be used to assess relationships between aquaporin localisation and post-translational regulations, such as phosphorylation state (**Figure 5E**); similar to the approaches taken by Prak et al. (2008) and Prado et al. (2019). This type of approach was used recently by Chen et al. (2021) to demonstrate PIP2 regulatory changes related to drought responses. External stimuli, like water limitation, often alters the phosphorylation state of plasma membrane proteins such as aquaporins (Orsburn et al., 2011). Regulation of environmental stress responses, and plant development, in many cases relies on phosphorylation-mediated regulation of membrane transport protein functions (Lin et al., 2015; Hsu et al., 2018). Lots of work lies ahead in determining the full complement of relevant phosphoproteins such as kinases, phosphatases, receptor-like kinases and other environmental stress-responsive interacting proteins and signals relevant to aquaporin regulation (Mattei et al., 2016; **Figure 5E**).

**Table 1:** Examples of associations between phosphatase activity and aquaporin regulation.

Species, AQP	Category (name)	Evidence	Observations	References
*Camelina sativa*, PIP2;1 & PIP2;6	PPP	Okadaic acid treatment in *Xenopus* oocytes ^a^	Reduces H_2_O transport	(Jang et al., 2014)
*Glycine max*, NOD26	PPP	Okadaic acid treatment in *Xenopus* oocytes ^a^	Reduces H_2_O transport; salinity-responsive changes in phosphorylation	(Guenther et al., 2003)
*Poplar trichocarpa*, AQUA1	PPP	Okadaic acid treatment in *Arabidopsis* protoplasts ^a^	Increases the number and volume of AQUA1 cytosolic vesicles under Zn stress; changes membrane localisation	(Ariani et al., 2019)
*Solanum lycopersicum*, PIPs	PTP	Na_3_VO_4_ treatment on *Solanum* roots ^b^	Reduces root hydraulic conductivity under salt stress through decreased water transport	(Jia et al., 2020)
*Spinacia oleracea*, PM28a	PPP	Okadaic acid treatment in *Xenopus* oocytes ^a^	Reduces H_2_O transport	(Johansson et al., 1998)
*Tulipa gesnerina*, putative PIP	PPP (PP2A)	*In vitro* phosphorylation assay	Reduces H_2_O transport; involved in temperature-responsive flower opening & closing	(Azad et al., 2004a; Azad et al., 2004b)

a Okadaic acid is commonly considered a selective inhibitor of members of the PPP family, showing greatest sensitivity to PP2A and PP1 (Soldovieri, 2009).

b Na_3_VO_4_ is a competitive inhibitor of PTPs, as well as some alkaline phosphatases (from New England Biolabs).

Phosphatases are enzymes involved in removing a phosphate group from a protein. PPP (protein serine/threonine phosphatase); PTP (protein tyrosine phosphatase).

**Table 2:** Examples of associations between kinase activity and aquaporin regulation.

Species, AQP	Category (name)	Evidence	Observations	References
*Arabidopsis thaliana*, NIP1;1	CPK (CPK31)	Subcellular localisation & BiFC assay	Regulates arsenic tolerance	(Ji et al., 2017)
*Arabidopsis thaliana*, PIP2;1	RLK (Feronia)	*Xenopus* oocyte swelling assay	Reduces H_2_O transport; regulates cell growth	(Bellati et al., 2016)
*Arabidopsis thaliana*, PIP2;1	STK (OST1/ SnRK2.6)	*In vitro* phosphorylation assay	Increased H_2_O transport; ABA-induced stomatal closure	(Grondin et al., 2015)
*Arabidopsis thaliana*, PIP2;1	RLK (BAK1)	*In vitro* phosphorylation assay	Increased H_2_O and H_2_O_2_ transport; flg22-induced stomatal closure	(Rodrigues et al., 2017)
*Arabidopsis thaliana*, PIP2;4	RLK (SIRK1)	Protoplast swelling assay	Sucrose-responsive H_2_O transport	(Wu et al., 2013)
*Arabidopsis thaliana*, PIP3	RLK (BSK8)	Quantitative phosphoproteomics	Uncertain	(Wu et al., 2014)
*Arabidopsis thaliana*, PIP1;1 & PIP1;2	CPK (CPK7)	qPCR in WT and *cpk7 Arabidopsis* plants	Reduces cellular abundance of AQP protein	(Li et al., 2015)
*Arabidopsis thaliana*, TIP3;1	STK (PKA)	*In vitro* phosphorylation assay	Increases H_2_O transport	(Maurel et al., 1995)
*Camelina sativa*, PIP2;1 & PIP2;6	STK	K252a treatment in *Xenopus* oocytes	Reduces H_2_O transport	(Jang et al., 2014)
*Gentiana scabra*, PIP2;2 & PIP2;7	CPK (CPK16)	*In vitro* kinase and cell phosphorylation assays	Reversible flower opening	(Nemoto et al., 2022)
*Glycine max*, NOD26	CPK	Immunochemical isolation	Increases H_2_O transport; salinity-responsive changes in phosphorylation	(Weaver et al., 1991)
*Lens culinaris*, α-TIP	CPK	*In vitro* phosphorylation assay	Speculative involvement in seed germination	(Harvengt et al., 2000)
*Oryza sativa*, PIP1;1, PIP1;3 & PIP2;3	RLK (LP2)	Firefly luciferase complementation imaging assay	Regulation of drought tolerance	(Wu et al., 2015)
*Poplar trichocarpa*, AQUA1	PI3K	Wortmannin treatment in *Arabidopsis* protoplasts ^a^	Increases the number of AQUA1 cytosolic vesicles under Zn stress; changes membrane localisation	(Ariani et al., 2019)
*Solanum lycopersicum*, PIPs	PI3K	Wortmannin treatment in *Arabidopsis* protoplasts ^a^	Reduces root hydraulic conductivity under salt stress through decreased water transport	(Jia et al., 2020)
*Spinacia oleracea*, PM28a	STK	K252a treatment in *Xenopus* oocytes	Increases H_2_O transport	(Johansson et al., 1998)
*Spinacia oleracea*, PIP2;1	CPK	*In vitro* phosphorylation assay	Increases H_2_O transport	(Sjövall-Larsen et al., 2006)

a Wortmannin is a specific inhibitor of PI3Ks but may also be involved in inhibiting other kinases.

Protein kinases are key regulatory enzymes involved in attaching a phosphate group to a protein. CPK (Ca^2+^ dependent protein kinase); RLK (receptor-like kinase); STK (serine/threonine protein kinase); PI3K (phosphatidylinositol 3-kinase). Previous studies have reported kinases and phosphorylation sites relevant to the regulation of mammalian aquaporins (see Nesverova and Törnroth-Horsefield, 2019). There are types of kinases, like protein kinase A (PKA), that are relevant to regulation of both mammalian and plant aquaporins. PKAs activity is dependent on cellular levels of cyclic adenosine monophosphate (cAMP). cAMP is a derivative of adenosine triphosphate (ATP), many different organisms use cAMP as part of intracellular signal transduction and interplay between potassium, cAMP signaling, PKA, aquaporin regulation and cell water permeability has been reported in mammalian astrocyte cells (Song and Gunnarson, 2012).

Expressing aquaporins of interest in forms that control for post-translational modification effects in a range of genetic backgrounds can help to tease out what signals regulate aquaporin influence on water transport (**Figure 4**); similar to the approach used by Qing et al. (2016). It is relevant to use genetic backgrounds where endogenous aquaporin expression is restricted, and aquaporin candidates of interest are instead expressed through introduction of transgenes, where transgene expression is achieved in specific tissues and cell-types through careful selection of promoters (Israel et al., 2021). Plant material where candidate aquaporins of interest have been modified to influence their abundance, localisation and regulation could be subjected to changes in VPD, then leaf humidity measured over a time-course following the measurement approaches reported by Wong et al. (2022). To distinguish between root-associated aquaporin influence on transpiration and leaf water content versus shoot-associated aquaporin influence on leaf water content it may be relevant to use a grafting approach, or use cell-type-specific or inducible promoters (Christmann et al., 2007; Andersen et al., 2014; Reeves et al., 2022). For example, grafting root and shoot material from aquaporin loss-of-function mutants paired with loss-of-function mutants where the aquaporin function has been complemented by introduction of gene fragments enabling the expression of candidate aquaporins and testing whether different root genetic backgrounds influence leaf humidity regulation. In previous studies, inducible and mesophyll cell-specific promoters were used to investigate aquaporin roles in carbon dioxide transport in leaves (Flexas et al., 2006; Ermakova et al., 2021). In any approach it is necessary to factor in the influence of PTMs in regulating aquaporin function. These combinations of experimental approaches (**Figure 5**) would be expected to reveal which key aquaporins influence leaf humidity (**Figures 5A, B**), where they are located (**Figure 5C**), how they may be regulated (**Figures 5D, E**) and how manipulation of their function could influence plant regulation of leaf humidity (**Figure 5F**).

In summary, key opportunities for future research include (**Figure 5**):

- Narrowing down on the number of aquaporin candidates that might be involved in influencing changes in leaf humidity. This could involve measuring cell-specific aquaporin transcript abundance over a time-course following changes in VPD (Berkowitz et al., 2021; Chen et al., 2022), checking whether changes in respective aquaporin protein abundance do or do not correspond to transcript levels (Bellati et al., 2016; Prado et al., 2019; Uhrig et al., 2021; Yavari et al., 2022), and testing the sub-cellular localisation of the aquaporins (Tian et al., 2004; Li et al., 2011).
- Exploring whether PTMs influence the function of the aquaporin candidates in heterologous systems and *in planta*, and investigating the associated impact on cell signaling pathways and aquaporin permeability (Prak et al., 2008; Wu et al., 2015; Prado et al., 2019; Qiu et al., 2020; Chen et al., 2021).
- Resolving whether leaf humidity differs in plant material where candidate aquaporin function has been lost, increased or modified. This will involve factoring in the potential for the aquaporin candidates to be subject to PTMs influencing their function (Qing et al., 2016; Wong et al., 2022; Zhang et al., 2023).

## Concluding remarks and perspectives

Agricultural productivity loss due to insufficient soil and atmospheric water availability is a major threat to food security (Zhou et al., 2019; Qing et al., 2022). Manipulation of aquaporin function is an example of one key target, among many options, for engineering crops to improve their tolerance to growing environments with sub-optimal water availability (Bowerman et al., 2023; De Rosa et al., 2023).

Studying and engineering aquaporin function in crops is challenging due to the large numbers of aquaporin isoforms present and because aquaporin function and regulation is complex. Aquaporins can transport many different molecules, and influence signaling pathways, and their localisation, gating and permeability are dynamic. However, optimising the regulation of aquaporins can contribute to adapting crop plants to environmental conditions where water availability is sub-optimal by enabling crops to have enhanced control of the flux of water, osmolytes and signaling molecules relevant to rapidly adjusting tissue hydration.

Resolving how different aquaporins contribute to different hydraulic features in plants such as leaf humidity regulation, stomatal function, root-to-shoot solute transport, root solute uptake, cell and tissue osmoregulation, and stress signaling is expected to position the field with key information needed to devise strategies for improving crop productivity in challenging environmental conditions.

## Author contributions

All authors listed have made a substantial, direct, and intellectual contribution to the work, and approved it for publication.
